# Off-pump bidirectional Glenn through right anterior thoracotomy

**DOI:** 10.5935/1678-9741.20150047

**Published:** 2015

**Authors:** Maximo Guida, Andrea Lo Cascio, Gustavo Guida, Gabriel Guida, Estefania De Garate, Manuel Vasquez, Fernando Prieto, Miriam Pecchinenda

**Affiliations:** 1 Fundacardio Foundation, Valencia, Venezuela.

**Keywords:** Cardiopulmonary Bypass, Anastomosis, Surgical, Thoracotomy, Heart Defects, Congenital, Cyanosis

## Abstract

The Glenn operation involving anastomosis of the superior vena cava to the pulmonary artery has been performed for palliative operations of many cyanotic congenital heart diseases in addition to the single ventricle since the 1960s. The classic procedure is done via median sternotomy and cardiopulmonary bypass. The benefits of this procedure without the use of cardiopulmonary bypass remain mixed within reported series. Cases using this approach and off-pump technique together in Latin-America have not yet been reported in the scientific literature.

## Abstract (pt)

A operação de Glenn envolvendo anastomose da veia cava superior à artéria pulmonar foi realizada como procedimento paliativo de muitas doenças cardíacas congênitas cianóticas, além do ventrículo único desde os anos 1960. O procedimento clássico é feito por esternotomia mediana e circulação extracorpórea. Os benefícios deste procedimento sem o uso de circulação extracorpórea permanecem incertos dentro da série relatada. Casos utilizando conjuntamente esta abordagem e a técnica sem circulação extracorpórea na América Latina ainda não foram relatados na literatura científica.

**Table:** 

**Abbreviations, acronyms & symbols**	
ABG	Arterial blood gas
ACT	Activated coagulation time
BGP	Bidirectional Glenn procedure
CPB	Cardiopulmonary bypass
ECG	Ele ctro c ardi ogram
EtCO_2_	End-tidal carbon dioxide
PA	Pulmonary atresia
RPA	Right pulmonary artery
Sa0_2_	Oxygen saturation
SVC	Superior vena cava
VSD	Ventricular septum defect

## INTRODUCTION

The Glenn operation involving anastomosis of the superior vena cava to the pulmonary artery has been performed for palliative operations of many cyanotic congenital heart diseases in addition to the single ventricle since the 1960s^[1]^. The classic procedure is done via median sternotomy and cardiopulmonary bypass^[2,3]^ the benefits of this procedure without the use of cardiopulmonary bypass remain mixed within reported series because there are still controversial conclusions^[4-6]^.

Cases using this approach and off-pump technique together in Latin-America have not yet been reported in the scientific literature.

## CASE REPORT

A twenty-monthold male patient, body weighing 7 kg, diagnosed with pulmonary atresia (PA) ventricular septal defect (VSD) + pulmonary hypoplasia clinically presented with severe cyanosis, oxygen saturation (SaO_2_) of 35%, and history of generalized tonic-clonic seizure in several occasions, was referred to our service for a bidirectional Glenn procedure in order to improve his clinical condition; the Blalock-Taussing (BT) shunt was also considered, but our preference was the first procedure.

The Ethics Commettee aproval was granted either for the use of whole medical history contents and the scientific use of the data.

The patient's relatives also signed an informed consent for the surgery and the use of both pictures and content for scientific purpose.

Before surgery the patient had an angiogram with right heart catheterization to measure pulmonary artery pressure and also to determine whether the procedure was feasible.

The procedure was performed under general anesthesia. The intraoperative management included monitoring electrocardiogram (ECG), SaO_2_, end-tidal carbon dioxide (EtCO_2_). Arterial blood gas (ABG) was analyzed at the baseline after intubation and during the procedure. In addition pressure monitoring line was placed in the superior vena cava (SVC), an invasive arterial pressure line was placed in the femoral artery and central venous access was obtained using a trilumen catheter into the right femoral vein, the corporal temperature was monitorized by rectal probe and controlled at 36ºC with the use of a thermic mattress as well as the operation room's temperature, all this as part of our usual surgical protocol.

**Fig. 1:**
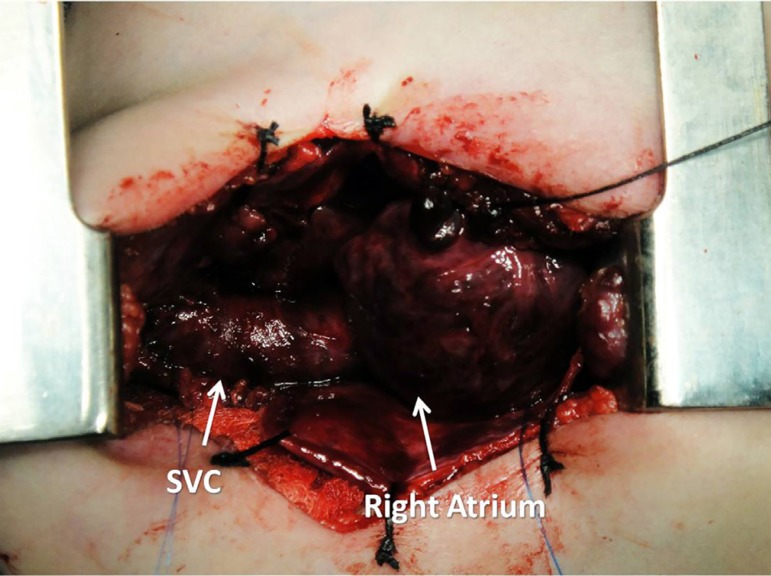
Approach - The picture presents first exposure of the superior vena cava and the right atrium.

A right anterior thoracotomy was performed in the 4^th^intercostal space; after the pleural cavity was opened the right lung was partially retracted with the use of lap sponges, checking the oxygen saturation and hemodynamic stability (Figure 1).

Circumferential control was gained around the entire length of the SVC, exposing this vessel completly by dissecting from the adjacent tissues. The azygous vein was ligated at this stage, this was to ensure non steal phenomenon from the SVC to inferior vena cava through this vein. The right pulmonary artery was exposed, and circumferential control was gained around the right main pulmonary artery as well as the hilar branches. Intraoperative pressure of the right pulmonary artery (RPA) was measured for the feasibility of the procedure.

**Fig. 2:**
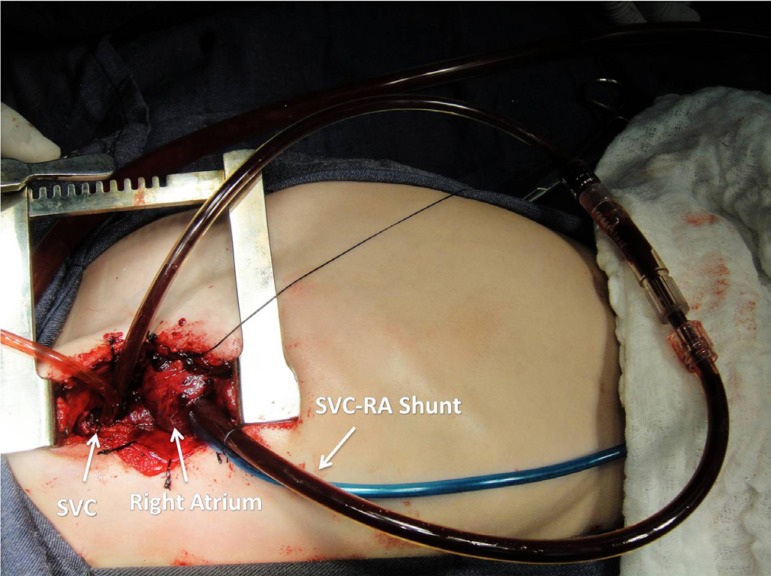
SVC-RA shunt - In this picture the SVC-RA shunt is already set in position and working while the SVC-RPA anastomosis is performed. SVC-RA=superior vena cava and the right atrium; SVC-RPA=superior vena cava and right pulmonary artery

Two purse-string-sutures with 5-0 polipropilene were performed, one at the proximal side of the SVC and the other at the right atrial appendage. The patient was systemic heparinized with 300 units/kg to maintain activated coagulation time (ACT) above 250. A 12F right-angle cannula was placed high on the SVC and a 12F straight cannula was placed in the right atrium. These cannulas were de-aired and hooked together to create a venoatrial shunt and allow drainage of the upper body while the proximal SVC was occluded (Figure 2).

**Fig. 3:**
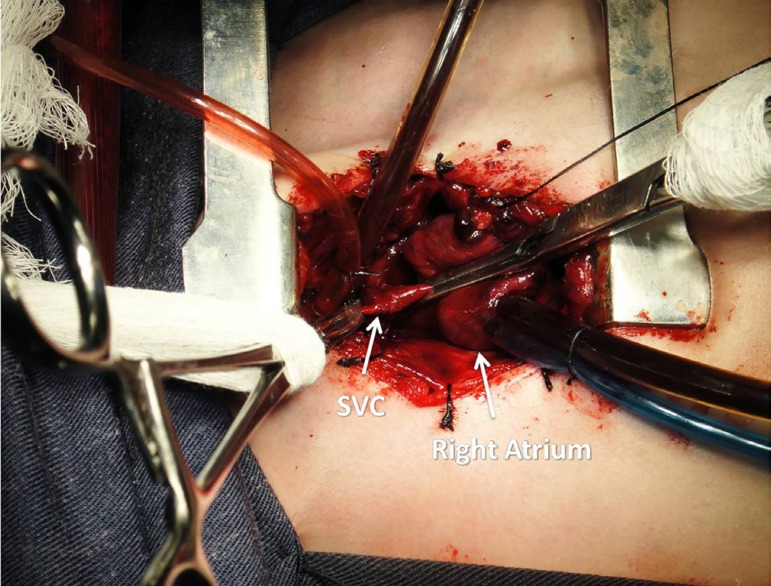
SVC clamp - The picture presents the SVC clamped before the SVC-RPA anastomosis. SVC=superior vena cava; SVC-RPA=superior vena cava and right pulmonary artery

**Fig. 4:**
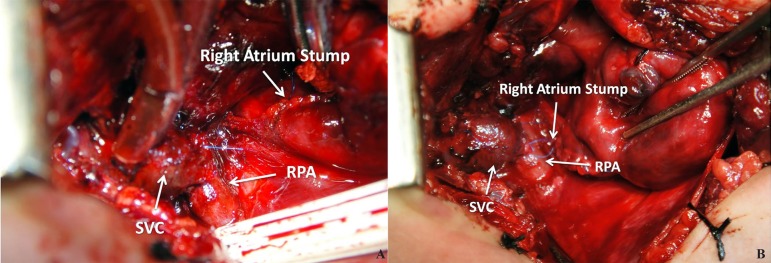
Final result - It is shown the SVC-RPA anastomosis and the RA stump in this picture. SVC-RPA=superior vena cava and right pulmonary artery; RA=right atrium

The pulmonary artery was temporarily occluded using a partial clamp, to ensure acceptable oxygen saturations (maintained between 50-60%) and hemodynamic stability (Figure 3). The SVC was clamped and sectioned distally; the stump was oversewed using two layers of 6-0 polipropilene (Figure 4A).

The SVC was anastomosed to the RPA using continuous 6-0 polipropilene suture; the clamps were removed and hemostasis achieved (Figure 4B). The shunt between the SVC and the right atrium was removed and the heparin reverted with protamine, finally the surgical incision was closed conventionally, the right pleura was drained using a single Blake system chest drain, connected to a conventional vacuum system. The patient was extubated in the operation room and the O_2_ saturation raised to 90%, the patient was then transferred to intensive care unit in stable general and hemodynamic conditions.

The patient had a satisfactory recovery and was discharged on the 4^th^ postoperative day in good general conditions.

## DISCUSSION

The bidirectional Glenn procedure (BGP) can be performed via median sternotomy or anterior right thoracotomy, furthermore the use of cardiopulmonary bypass or a temporary shunt between SVC and the right atrium can also be considered. The decision about the approach and strategy is mainly based on the surgical team experience, patient's condition and the perioperative risk.

Many published studies have shown good results when the cardiopulmonary bypass (CPB) is avoided^[5,7]^; furthermore the original surgical technique described by Glenn in 1958 was performed through a thoracotomy and without CPB^[8]^.

One of the concerns of using the veno-atrial shunt instead of CPB is the risk of inadequate brain protection, due to its flow capacity and the clamping time of the SCV^[5,7]^. One of the measures to improve the brain protection is clamping the SCV underneath the insertion of the azygos vein, as well as the use of a dopamine infusion and a good general circulation volume^[5,7]^, all this to achieve a high transcraneal pressure gradient. The use of Fowler position in the operation table is also beneficial.

Some authors have excluded patients with O_2_ saturation under 65%, because they considered it increases the risk for operative mortality^[9]^. The case we are presenting had an O_2_ saturation of 35% which according with the literature would be considered as a very high risk patient for this procedure. However, the postoperative course confirmed the feasibility of the technique.

Avoiding the use of cardiopulmonary bypass has multiples advantages, especially in low weight patients, decreasing the deleterious effects produced on the rest of the organs and vital systems^[10]^. The right anterior thoracotomy approach represents a good choice for selected patients, leaving the median sternotomy available for future interventions.

In this case the postoperative course was highly satisfactory and after ten months after surgery the patient remains in excellent clinical conditions, maintaining O_2_ saturation in ambient air above 85%.

## CONCLUSION

This case report showed a really good evolution combining the off-pump bidirectional Glenn procedure with a right anterior thoracotomy approach. A larger series of patients is needed to evaluate the proper indications and results of this technique. In our service now we are selecting patients not previously operated, without adherences and without a BT shunt sutured in the right pulmonary artery, meanwhile the learning curve would overpassed.

We consider this proposed technique as a good and safe option in a favorable patients avoiding median sternotomy and extracorporeal circulation with all of the related problems when possible achieving excellent results.

**Table:** 

**Authors’ roles & responsibilities**	
MG	Conception and design; manuscript writing or critical review of its content
ALC	Manuscript writing or critical review of its content
GG	Conception and design; manuscript writing or critical review of its content
GG	Manuscript writing and critical review of its content
EG	Conception and design
MV	Manuscript writing and critical review of its content
FP	Conception and design
MP	Conduct of operations and experiments
